# Common pathophysiology for ANXA11 disorders caused by aspartate 40 variants

**DOI:** 10.1002/acn3.51731

**Published:** 2023-01-18

**Authors:** Daniel Natera‐de Benito, Jonathan Olival, Carla Garcia‐Cabau, Cristina Jou, Mònica Roldan, Anna Codina, Jessica Expósito‐Escudero, Cristina Batlle, Laura Carrera‐García, Carlos Ortez, Xavier Salvatella, Francesc Palau, Andrés Nascimento, Janet Hoenicka

**Affiliations:** ^1^ Neuromuscular Unit, Department of Neurology Hospital Sant Joan de Déu Barcelona 08950 Spain; ^2^ Applied Research in Neuromuscular Diseases Institut de Recerca Sant Joan de Déu Barcelona 08950 Spain; ^3^ Laboratory of Neurogenetics and Molecular Medicine – IPER Institut de Recerca Sant Joan de Déu 08950 Barcelona Spain; ^4^ Institute for Research in Biomedicine (IRB Barcelona) The Barcelona Institute of Science and Technology Barcelona 08029 Spain; ^5^ Department of Pathology Hospital Sant Joan de Déu Barcelona 08950 Spain; ^6^ Confocal Microscopy and Cellular Imaging Unit Institut de Recerca Sant Joan de Déu Barcelona 08950 Spain; ^7^ Department of Genetics and Developmental Medicine – IPER Hospital Sant Joan de Déu Barcelona 08950 Spain; ^8^ Center for Biomedical Research Network on Rare Diseases (CIBERER) ISCIII Barcelona Spain; ^9^ ICREA Barcelona 08010 Spain; ^10^ Division of Pediatrics, Faculty of Medicine and Health Sciences University of Barcelona Barcelona 08007 Spain; ^11^ ERN ITHACA Barcelona 08950 Spain

## Abstract

**Objective:**

Mutations in *ANXA11* cause amyotrophic lateral sclerosis (ALS) and have recently been identified as a cause of multisystem proteinopathy and adult‐onset muscular dystrophy. These conditions are adult‐onset diseases and result from the substitution of Aspartate 40 (Asp40) for an apolar residue in the intrinsically disordered domain (IDD) of ANXA11. Some ALS‐related variants are known to affect ANXA11 IDD; however, the mechanism by which the myopathy occurs is unknown.

**Methods:**

Genetic analysis was performed using WES‐trio. For the study of variant pathogenicity, we used recombinant proteins, muscle biopsy, and fibroblasts.

**Results:**

Here we describe an individual with severe and rapidly progressive childhood‐onset oculopharyngeal muscular dystrophy who carries a new *ANXA11* variant at position Asp40 (p.Asp40Ile; c.118_119delGAinsAT). p.Asp40Ile is predicted to enhance the aggregation propensity of ANXA11 to a greater extent than other changes affecting this residue. In vitro studies using recombinant ANXA11^p.Asp40Ile^ showed abnormal phase separation and confirmed this variant is more aggregation‐prone than the ALS‐associated variant ANXA11^p.Asp40Gly^. The study of the patient's fibroblasts revealed defects in stress granules dynamics and clearance, and muscle histopathology showed a myopathic pattern with ANXA11 protein aggregates. Super‐resolution imaging showed aggregates expressed as pearl strips or large complex structures in the sarcoplasm, and as layered subsarcolemmal chains probably reflecting ANXA11 multifunctionality.

**Interpretation:**

We demonstrate common pathophysiology for disorders associated with *ANXA11* Asp40 allelic variants. Clinical phenotypes may result from different deleterious impacts of variants upon ANXA11 stability against aggregation, and differential muscle or motor neuron dysfunction expressed as a temporal and tissue‐specific continuum.

## Introduction

Annexins are a large family of evolutionarily conserved proteins with a variable N‐terminal domain of member‐specific functions and a high structural homology in their C‐terminal core that allows Ca^2+^‐dependent membrane binding.^1^, ^2^ Although there is considerable functional diversity among annexins, most of their functions in cells are linked to their dynamic and reversible binding to membranes.^3^

Annexin A11 (ANXA11) is ubiquitously expressed and contains an unusually long N‐terminal, which is intrinsically disordered. ANXA11 is involved in cell division, Ca^2+^ signaling, and apoptosis.^4^ Recently, the ANXA11 N‐terminal domain was found to bind RNA and tether membraneless RNA granules to lysosomes via liquid–liquid phase separation (LLPS) to assemble stress granules (SGs).^5^, ^6^ Loss of SG homeostasis resulting in protein aggregation is a major contributor to both the initiation and progression of neurodegenerative diseases such as amyotrophic lateral sclerosis (ALS) and frontotemporal dementia (FTD).^7^ Indeed, mutations in the *ANXA11* gene cause ALS motor neuron disease 23 (ALS23, MIM #617839),^8^, ^9^ among them, p.Asp40Gly is related to late disease onset and classical ALS features.^8^ *ANXA11* mutations in the N‐terminal domain of the peptide, including p.Asp40Gly, enhance aggregation propensity and disrupt LLPS‐mediated SGs dynamics for long‐distance trafficking and mRNA delivery.

The phenotypic spectrum of *ANXA11* mutations has recently expanded into myopathies, with the description of Brazilian and Greek families that carry the N‐terminal variant p.Asp40Tyr. Some individuals of the Brazilian families had an adult‐onset inclusion body myopathy (IBM) manifested as part of a multisystem proteinopathy isolated or in combination with ALS or FTD,^10^ while all individuals from the Greek families had an adult‐onset myopathy without multi‐organ involvement or ALS.^11^ Mutations in genes encoding other proteins with intrinsically disordered domains (IDD) and functionally involved in SGs biology have been reported to cause myopathies. Specifically, mutations in *VCP*,^12^ *HNRNPA2B1* and *HNRNPA1*,^13^ *SQSTM1*,^14^ *MATR3*,^15^ and the aforementioned *ANXA11* ^10^ lead to adult‐onset myopathies with an accumulation of cytoplasmic aggregates in the muscle. In pediatric patients, however, only *HNRNPA2B1* is known to cause a childhood‐onset myopathy,^16^ resulting in a phenotype reminiscent of oculopharyngeal muscular dystrophy but with an exceedingly early onset.

Here we present a subject with a severe and rapidly progressive childhood‐onset oculopharyngeal muscular dystrophy who carries the new *ANXA11* variant p.Asp40Ile, which increases ANXA11 aggregation in vitro and muscle fibers, and causes defects of SGs dynamics in fibroblasts. This variant is actually in the same residue of p.Asp40Gly (ALS) and p.Asp40Tyr (IBM, ALS, FTD). Regardless of etiology, we provide evidence of the pathogenic relationship of *ANXA11* and *HNRNPA2B1*, and how alterations in LLPS and dynamics of SGs underlie the pathogenesis of *ANXA11*‐related disorders in which the Asp40 residue is involved. Our findings extend the phenotypic spectrum of *ANXA11*‐related conditions to childhood‐onset progressive myopathies and demonstrate common pathophysiology for disorders associated with *ANXA11* Asp40 allelic variants.

## Materials and Methods

### Subject and clinical examinations

Data were collected following the ethics guidelines of Sant Joan de Déu Children's Hospital, Barcelona, and the ethical standards laid down in an appropriate version of the 1964 Declaration of Helsinki. Electrophysiological examination (nerve conduction studies and electromyogram), muscle MRI imaging, muscle biopsy, and blood samples for DNA analysis were collected for diagnostic purposes. Written informed consent and age‐appropriate assent for study participation were obtained from parents and the patient (protocols PIC‐30‐17 and PIC‐223‐19). Additional informed consent was obtained for muscle and skin biopsies.

### Molecular analyses

DNA was extracted from a peripheral blood sample using standard techniques. A whole‐exome sequencing‐trio (WES‐trio) was performed. To capture the coding regions, we used the Illumina DNA Prep with Enrichment exome capture (DNA Prep with Enrichment, Illumina, San Diego, CA). Sequencing with 2 × 121 bp read lengths was performed by loading the flow cell on the Illumina NextSeq 500 System. Exome sequencing data were processed through a pipeline based on Picard (https://broadinstitute.github.io/picard/) and mapping was done using the BWA aligner (http://bio‐bwa.sourceforge.net/bwa.shtml)^17^ to the human genome build 37. Variants were called using four different programs: SAMtools version 1.5 (Wellcome Trust Sanger Institute, Cambridge, UK), GATK Haplotype Caller package version 3.7 (Broad Institute, Cambridge, MA), FreeBayes version 1.1.0 (Boston College, Boston, MA), and VarScan version 2.4.0 (Washington University, St. Louis, MO). The pathogenicity classification of genetic variants was determined by the ACMG guidelines (performed using VarSome, https://varsome.com, last accessed November 6, 2022).^18^, ^19^ We further assessed pathogenicity using MutationTaster,^20^ SIFT,^21^ PROVEAN,^22^ PolyPhen2,^23^ FATHMM^24^ and CADD.^25^ To evaluate the frequencies of the variants in global populations (last accessed November 6, 2022) we used the Genome Aggregation Database (gnomAD),^26^ ClinVar^27^ and the CIBERER‐Collaborative Spanish Variant Server (CSVS).^28^ PCR and Sanger sequencing were used for variant validation.

### Muscle biopsy, histological, immunohistochemical and immunofluorescence studies

Snap‐frozen muscle biopsy specimens were sampled from right deltoids and processed according to standard muscle histopathological techniques.^29^ Histological and histochemical techniques were performed on 8 μm transversely orientated cryosections of skeletal muscle, including hematoxylin–eosin, modified Gomori's trichrome, nicotinamide adenine dinucleotide (NADH), succinate dehydrogenase (SDH), cytochrome *c* oxidase (COX), periodic acid‐Schiff, and Oil Red O, as described previously.^29^ Immunohistochemical studies were performed following conventional protocols and using specific antibodies (Table S1).

For immunofluorescence staining the muscle sample was cut into 10 μm thick sections, fixed (4% paraformaldehyde at room temperature (RT) for 7 min), rinsed with PBS‐Tween (0.5%), and blocked with 1% Tween‐ 8% BSA‐ 10% donkey serum in PBS for 1 h at RT. Antibody incubation was performed overnight at 4°C using the following primary antibodies: rabbit polyclonal α‐ANXA11 (1:1000), mouse monoclonal α‐hnRNPA2B1 (1:50) and mouse monoclonal α‐SQSTM1 (1:20). After primary antibody incubation, sections were washed in PBS‐tween‐triton and incubated for 3 h at RT with the secondary antibodies α‐mouse Alexa Fluor 488 and α‐rabbit Alexa Fluor 594 (Table S1). For counterstaining of nuclei, sections were incubated 5 min with DAPI (1:5000, Thermo Fisher Scientific, Inc.) and rinsed in PBS‐Tween‐triton before mounting onto Fluoromount G (Thermo Fisher Scientific, Inc.).

### Muscle ultrastructural analysis

For electron microscopic studies, small fragments of muscle were fixed in 2.5% glutaraldehyde solution in cacodylate buffer pH 7.4 and postfixed in 1% osmium tetroxide solution in the same buffer. After dehydration in a graded ethanol alcohol series and propylene oxide, specimens were embedded in Spurr resin. Semithin sections were stained with toluidine blue to identify appropriate areas. Ultrathin sections were contrasted with uranyl acetate and lead citrate. The sections were examined and photographed with transmission electron microscopy (JEOL model 1100). Electron micrographs were obtained using the Gatan Orius CCD camera (Olympus Soft Imaging Solutions, Münster, Germany).

### In silico studies of *ANXA11* variants

Sequence analysis of four ANXA11 variants (WT, p.Asp40Ile, p.Asp40Gly and p.Asp40Tyr) was carried out using PONDR VL‐XT for the sequence disorder predictions,^30^ Agadir algorithm for helicity prediction of the IDDs^31^ and Aggrescan for amyloid aggregation prediction.^32^

### Plasmid constructions for recombinant *ANXA11*

An insert containing the SUMO‐ANXA11 (1‐505) protein sequence was designed and ordered using the GeneArt software in Thermo Fisher Scientific. The insert was cloned in a pDONR211 vector and then subcloned into the expression vector pDEST17, resulting in the 6His‐SUMO‐*ANXA11* construct. p.Asp40Ile and p.Asp40Gly variants were obtained using the Q5 site‐directed mutagenesis kit (New England Biolabs, Ipswich, MA, USA).

Recombinant proteins were expressed in transformed *Escherichia coli* B834 cells. The cells were grown in LB medium at 37°C until an OD = 0.6, and induced with 0.1 mM IPTG overnight at 25°C. The cultures were then centrifuged for 30 min at 4000 rpm and the cells were resuspended in lysis buffer (PBS, 1 mM DTT, 0.05% NaN_3_, PIC, and PMSF, at pH 7.4). The cells were lysed by sonication and centrifuged for 30 min at 20,000 rpm. The pellet was washed twice with wash buffer (PBS, 1 mM DTT, 500 mM NaCl, 1% TritonX‐100, PIC, PMSF, DNAse, and RNAse, at pH 7.4). The pellet was resuspended in resuspension buffer (20 mM Tris–HCl, 8 M urea, 50 mM NaCl, 1 mM DTT, pH 8.0) and centrifuged for 30 min at 20,000 rpm. The sample was injected in a Ni^2+^ affinity column and eluted with a gradient from 0 to 500 mM imidazole (at 4°C and without urea for the cases in which the protein was present in the wash buffer supernatants, and at RT and with 8 M urea for the cases in which the protein was present in the resuspension buffer supernatant). The samples in urea were then stepwise dialyzed from 8 to 0 M urea. The 6His‐SUMO tag was cleaved with SUMO protease through dialysis for 2 h at 4°C in cleavage buffer (20 mM Tris–HCl, 50 mM NaCl, 1 mM DTT, pH 8.0). 8 M urea was added to the sample and injected into a nickel column to remove the cleaved tags. The reverse Ni^2+^ column was run at RT with the 8 M urea buffers containing 0 and 500 mM imidazole. The flow‐through was injected in a size exclusion superdex200 (GE Healthcare), running in 20 mM HEPES, 200 mM NaCl, 1 mM TCEP, 0.05% NaN_3_, pH 7.4 buffer. The fractions with protein were pooled and concentrated to 25–50 μM, fast‐frozen in liquid nitrogen and stored at −80°C.

### Sample preparation for in vitro LLPS experiments

All samples were prepared on ice as follows. First, a buffer stock solution consisting of 20 mM HEPES, 1 mM TCEP and 0.05% NaN_3_ was pH adjusted to 7.4 and filtered using 0.22 μm sterile filters (Buffer Stock). A 1 M NaCl solution in the same buffer was also pH adjusted to 7.4 and filtered (Salt Stock). Then, the protein samples were thawed from −80°C on ice, pH adjusted to 7.4, and centrifuged for 5 min at 15,000 rpm. The supernatant (Protein Stock) was transferred to a new Eppendorf tube and the protein concentrations were determined by their absorbance at 280 nm using 42,750 M^−1^ cm^−1^ as the extinction coefficient value using a Cary100 ultraviolet–visible spectrophotometer. The indicated samples were prepared by mixing the right amounts of Buffer Stock, Protein Stock, and Salt Stock to reach the desired final protein and NaCl concentrations.

### Apparent absorbance in function of temperature

The absorbance of the samples was measured at 350 nm (A_350 nm_) using 1 cm pathlength cuvettes and a Cary100 ultraviolet–visible spectrophotometer equipped with a multicell thermoelectric temperature controller. The temperature was increased progressively from 10 to 40°C at a ramp rate of 1°C/min. The cloud temperatures (Tc) were determined as the maximum of the derivatives of the curves, and the absorbance increase (ΔA_350 nm_) represents the difference between the maximum and the minimum absorbance values of the samples during the temperature ramp.

### Differential interference contrast microscopy

1.5 μL of the sample was deposited in a sealed chamber comprising a slide and a coverslip sandwiching double‐sided tape (3M 300 LSE high‐temperature double‐sided tape of 0.17 mm thickness). The used coverslips were previously coated with PEG‐silane following the published protocol.^33^ The DIC images were taken using an automated inverted Olympus IX81 microscope with a 60×/1.20 water UPlan SAPo objective using the Xcellence rt 1.2 software.

### Cell culture, treatments, and immunofluorescence

Fibroblasts from healthy controls were obtained from Sant Joan de Déu Children's Hospital Biobank. These cells were cultured in Dulbecco's modified Eagle medium (Sigma‐Aldrich, St. Louis, MO, USA) supplemented with 10% v/v fetal bovine serum (FBS, Gibco, Thermo Fisher Scientific, Inc.), 2 mmol/L l‐glutamine (Sigma‐Aldrich St. Louis, MO, USA), and 100 mg/mL penicillin–streptomycin (Sigma‐Aldrich St. Louis, MO, USA). Cell culture was maintained at 37°C in a 5% co_2_ humidified atmosphere. To induce stress granules assembly, fibroblasts were incubated at 37°C in a 5% co_2_ with 0.5 mM of sodium arsenite (NaAsO_2_) from Honeywell Fluka (Morristown, NJ, USA) as a stressor for 1 h.

For immunofluorescence studies, fibroblasts were seeded onto glass coverslips and fixed in 4% paraformaldehyde for 20 min. Permeabilization was performed using 0.2% TritonX‐100 diluted in PBS for 30 min at RT. Cells were blocked in PBS, 1% BSA, and 4% goat serum for 1 h at RT. The following primary antibodies were used and incubated overnight at 4°C: α‐ANXA11 (1:700), α‐hnRNPA2B1 (1:100), and α‐G3BP1 (1:400) (Table S1). After washing in PBS, cells were incubated at RT for 2 h in the dark using the secondary antibodies α ‐mouse Alexa Fluor 488 and α‐rabbit Alexa Fluor 594. Coverslips were mounted using Fluoromont‐G with DAPI.

### Quantitative real‐time RT‐PCR

Total RNA was extracted from control and patient fibroblasts using Trizol (TRItidy G™ AppliChem GmbH, Darmstadt, DE) following conventional protocol. Reverse transcription was performed using the Maxima First Strand cDNA Synthesis kit (Thermo Fisher Scientific, Inc.) according to the manufacturer's instructions. Quantitative RT‐PCR reactions were carried out three times in triplicate on a QuantStudio 6 Real‐Time PCR System using PowerUp SYBR Master Mix (Sigma‐Aldrich, St. Louis, MO, USA) and custom primers (Table S2) for *ANXA11* and *HNRNPA2B1*. The reaction conditions were as follows: 95°C for 10 min, then 40 cycles of 95°C for 15 sec, 59°C for 1 min, and 72°C for 45 sec, followed by one cycle of 95°C for 15 sec, 60°C for 1 min, and 95°C for 15 sec. *ANXA11* and *HNRNPA2B1* gene expression was quantified by the standard curve method^34^ and samples were normalized to glyceraldehyde‐3‐phosphate dehydrogenase (*GAPDH*) housekeeping gene.

### Western blot analysis

For total protein extraction in muscles, we used RIPA lysis buffer (Bio Basic, ON, Canada). The sample lysate was homogenized using pellet pestles and incubated for 5 min on ice. Next, the samples were centrifuged at 4°C at 18,000 *g* for 5 min to recover the supernatant (soluble fraction). In fibroblasts, extraction of the protein soluble fraction was performed with lysis buffer (10 mM Tris [pH 7.4]; 1% Triton X‐100; 150 mM NaCl; 10% glycerol) with added complete 1X protease inhibitor cocktail (Roche, Basilea, Switzerland) and PMSF 0.1 mM (Sigma‐Aldrich St. Louis, MO, USA). The cell lysate was subsequently sonicated for 8 sec (amp: 21%) and incubated for 1 h on ice. After incubation, samples were centrifuged at 4°C at 15,000 *g* for 20 min to recover the supernatant (soluble fraction). The BCA Protein Assay Kit (Pierce; Thermo Fisher Scientific, Inc.) was used to quantify the protein extraction in both fibroblasts and muscle. Western blots were performed following conventional protocols. Cell soluble fractions were separated with 10% SDS‐PAGE gels for analysis and transferred onto PVDF membrane (Millipore, Sigma‐Aldrich St. Louis, MO, USA). Membrane blocking was performed with 5% skim milk for 1 h. The membranes were subsequently incubated with the specific primary antibodies α‐ANXA11 (1:4000), α‐hnRNPA2B1 (1:500) at 4° overnight and in agitation. The next day, after being washed with TBST‐1X buffer, the membrane was incubated with a goat α‐mouse peroxidase‐conjugated IgG (1:10,000) and goat α‐rabbit peroxidase‐conjugated IgG (1:10,000) as secondary antibodies for 2 h at RT. Protein bands were detected using an ECL kit detection system (Cytiva Amersham Pl, UK). Semi‐quantification was performed using ImageJ version 1.46 (NIH).

### Confocal and super‐resolution imaging

Confocal and super‐resolution microscopy analysis was performed by Leica TCS SP8 equipped with a white light laser, HyVolution and Hybrid spectral detectors (Leica Microsystems GmbH, Mannheim, Germany). The confocal images of both muscle biopsies and fibroblast cultures were acquired using an HC × PL APO 20×/0.75 dry and an HC × PL APO 63×/1.4 oil immersion objectives. The super‐resolution images were acquired using an HC × PL APO 100×/1.4 oil immersion objective, the HyD detector, and HyVolution. DAPI was excited with a blue diode laser (405 nm) and detected in the 425–480 nm. Antibodies bond to Alexa Fluor 488 were excited with an argon laser (488 nm) and detected in the 505–550 nm. Antibodies bond to Alexa Fluor 594 were excited with a white light laser (594 nm) and detected in the 610–795 nm. In fibroblasts and muscular biopsy, *Z* stacks of 10–15 sections were acquired every 0.5–0.8 μm along with the sample thickness. Appropriate negative controls were used to adjust confocal settings to avoid non‐specific fluorescence artifacts. Image pixel was 8 bits depth and diameter pinhole diameter was 1 AU. To compare the confocal data, identical confocal settings were used for image acquisition of different experiments. Maximum intensity projections were performed using LAS X software and fluorescence quantification by the ImageJ software (National Institutes of Health, Bethesda, MD, USA).

For the super‐resolution images in muscle biopsies, *Z* stacks were acquired every 0.35 μm along with the fiber thickness. Image deconvolution was performed with Huygens Professional software v17.10.0p7 64b (SVI, Leiden, The Netherlands). Stacks were reconstructed and visualized as three‐dimensional (3D) volumes with Imaris software (Bitplane). ANXA11 area analysis was done with Imaris software using the Surface function, and the aggregate area was measured using the Surface Statistics function.

### Stress granules quantification

Endogenous G3BP1‐positive stress granules (SGs) in control and patient fibroblasts were quantified following stress induction (0.5 mM sodium arsenite for 1 h) for assembled SGs and after recovery (fresh medium for 1.5 h) for the remained SGs. 100 cells per condition were quantified blindly per independent experiment (*n* = 3).

### Statistical analysis

All data are expressed as means ± SEM, column bars or scatter plots graphs, showing the error bars and the individual values, respectively. *p* values are indicated by asterisks: *p < 0.05, **p < 0.01, and ***p < 0.001. The parametric unpaired *t*‐test was used to evaluate the following: differences in intensity of fluorescence of ANXA11 and hnRNPA2B1 in fibroblasts in basal, stress, and recovery conditions; assess the mRNA expression to *GAPDH* of *ANXA11* and *HNRNPA2B1* in basal and stress conditions; to compare the percentage of assembled and remained ANXA11+ G3BP1+ stress granules, in fibroblasts in stress and recovery conditions, respectively. One sample *t*‐test of transformed values was used to measure the protein expression levels of ANXA11 and hnRNPA2B1 in fibroblast in basal and stress conditions (normalized values were compared against control basal) and ANXA11 and hnRNPA2B1 in muscle fibers in basal condition. Statistical computing and graphs were performed using GraphPad Prism version 8.0.1 (GraphPad Software, Inc., La Jolla, CA).

## Results

### Clinical features were reminiscent of oculopharyngeal muscular dystrophy

**Figure 1:**
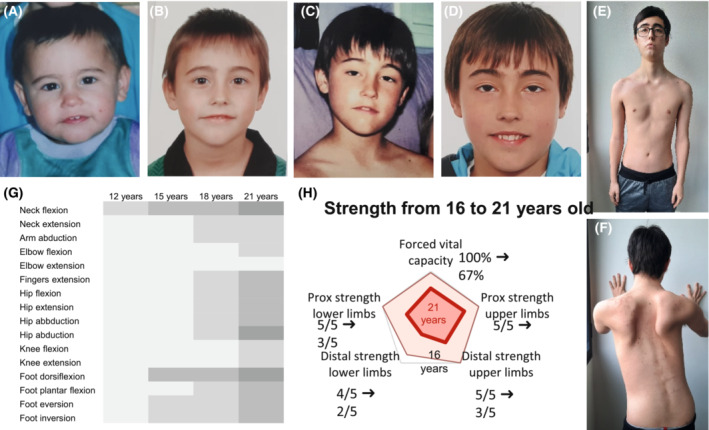
Clinical features of the index case and pattern of muscles weakness. Pictures of the index case show the absence of ptosis at preschool years (A,B); evident ptosis at middle childhood (C) and early adolescence (D) respectively; facial weakness and scapular winging in late adolescence (E,F). Heat map showing the evolution of the pattern of muscle weakness from childhood to late adolescence (G). Radar chart showing the evolution of forced vital capacity and weakness over the years: 16 years in light red and 21 years in dark red (H). The patient gave informed consent to use photographs showing his face. The Medical Research Council (MRC) Scale for Muscle Strength was used to assess muscle strength from Grade 5 (normal; in white) to Grade 0 (no visible contraction; in dark gray).

The proband is a Caucasian Spanish male in his 20 sec with a negative family history of neuromuscular conditions. Progressive ptosis was noted in middle childhood, after some uneventful first years of life, including normal psychomotor development. Opthalmoparesis and neck flexor weakness appeared later but also in the first decade of life. From the age of 14, progressive axial and facial weakness was detected, as well as scapular winging and proximal and distal weakness, more pronounced in the lower extremities. Bulbar symptoms, including dysphonia and dysphagia, were first detected in middle adolescence and have progressed since then, as has limb weakness. Currently, in his 20 sec, the individual has difficulty walking independently and is unable to climb stairs or rise from the floor unassisted. Detailed clinical information and pattern of weakness can be found in Figure 1 and Table S3.

**Figure 2:**
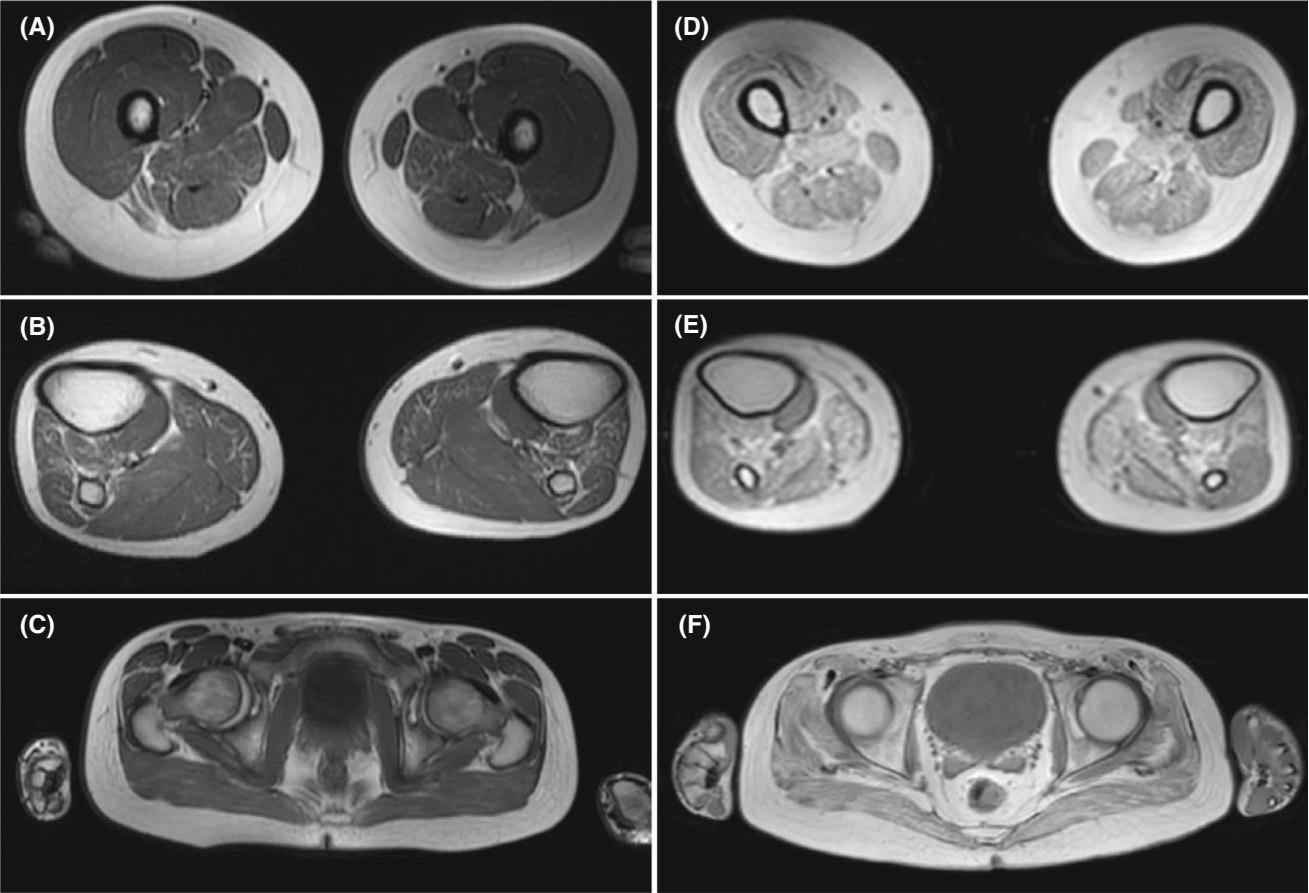
Whole‐body muscle MRI findings of the index case in adolescence. T1‐weighted MRI sequences of lower limb muscles in middle (A–C) and late (D–F) adolescence showed striking progression of fatty infiltration in all muscles. In middle adolescence the fatty infiltration on thighs (A) was observed on adductor magnus; while on calves (B) was more marked on tibialis anterior, extensor digitorum longus, tibialis posterior, and medial gastrocnemius. Muscles of the pelvis (C) were spared. At his 20 sec (D–F) the fatty infiltration and muscle atrophy have significantly progressed on pelvic musculature (psoas, gluteus) and anterior and posterior compartments of both thighs and legs, but also in erector spinae, paravertebral, latissimus dorsi, serratus, abdominal wall muscles, pectoralis, rotator cuff muscles, and biceps. The popliteus muscle (E) was among the very few relatively spared muscles.

Creatine kinase (CK) was normal until early adolescence and progressively increased up to 950 IU in late adolescence (last determination). Nerve conduction studies in the median, sural, ulnar, and peroneal nerves were normal. Electromyogram of the biceps in middle childhood and of the tibialis anterior, vastus medialis, rectus abdominis, and tibialis anterior in late adolescence revealed polyphasic, short‐duration, low‐amplitude motor unit action potentials, thus indicating a primary myopathy. Repetitive stimulation at the tibialis anterior and abductor digiti minimi revealed no neuromuscular junction abnormalities. Whole‐body muscle MRI in middle adolescence showed muscle atrophy and a mild focal fatty replacement of muscle in calfs (soleus, gastrocnemius, tibialis anterior, and tibialis posterior muscles), posterior thigh compartments, and teres minor muscles, while in late adolescence, a striking progression of muscle atrophy and fatty infiltration on virtually all the muscles, but especially in lower limbs was found (Fig. 2). Sitting and supine forced vital capacity in late adolescence were 81% and 67%, respectively. This decrease of 14% in forced vital capacity from sitting to the supine position is suggestive of diaphragmatic weakness. The cardiological study performed in late adolescence was normal.

### Genetic findings identified a new change in aspartate 40 of ANXA11

**Figure 3:**
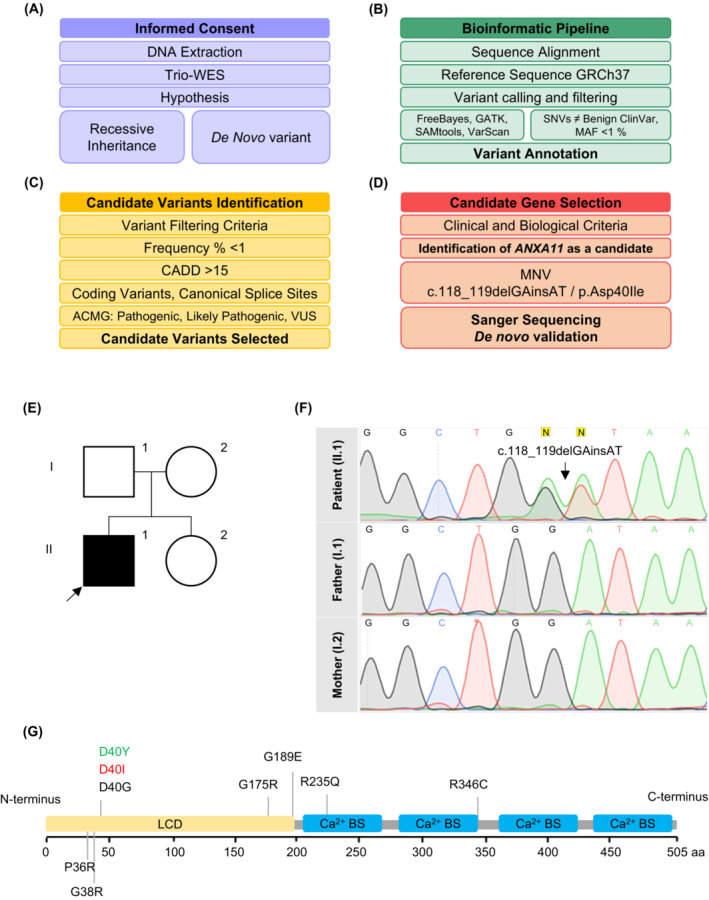
WES‐trio study and bioinformatics pipeline identified a novel disease mutation in *ANXA11*. (A) Initial process of family recruiting, WES‐trio, and hypothesis development. (B) Bioinformatics pipeline for genetic raw data analysis. (C) Filtering criteria for candidate variants analysis and identification. (D) Identified candidate variant was selected for further analysis. (E) Pedigree of the family. (F) Electropherogram showing the *ANXA11* c.118_119delGAinsAT de novo variant in the index case, and the absence in their parents. (G) ANXA11 protein structure and domains showing *ANXA11* gene variants associated with ALS (black), adult‐onset inclusion body myopathy (green) and early‐onset oculopharyngeal muscular dystrophy (red). ACMG, American College of Medical Genetics; CADD, Combined Annotation Dependent Depletion; MAF, Minor Allele Frequency; MNV, Multiple Nucleotide Variant; SNV, Single Nucleotide Variant; VUS, Variant of Uncertain Significance.

Whole‐exome sequencing of the patient and his parents (WES‐trio) identified a new deletion/insertion of two consecutive nucleotides (c.118_119delGAinsAT) in the *ANXA11* gene (MIM*602572; NM_145868.2). The bioinformatics pipeline to identify disease‐causing variants is shown in Figure 3A. Segregation analysis revealed de novo inheritance of the variant, which substitutes an evolutionarily conserved Aspartate (Asp) residue in the IDD of ANXA11 peptide with an Isoleucine (Ile) residue (p.Asp40Ile) (Fig. 3B–D). Based on ACMG guidelines and computational pathogenicity prediction tools, p.Asp40Ile was classified as likely pathogenic (PS2, PM2, PM5, and PP3 criteria). The variant would be reclassified as pathogenic after PS3 confirmation (well‐established in vitro and in vivo functional studies support a damaging effect on the gene or protein). Additionally, the variant p.Asp40Ile is absent in control populations (Table S4). Because of the report of variants in *HNRNPA2B1* in patients with oculopharyngeal muscular dystrophy,^35^ we reanalyzed this gene in the patient. We did not find any variant in the *HNRNPA2B1* gene. Therefore, we considered that the *ANXA11* p.Asp40Ile substitution was the candidate disease‐variant in our patient.

### Histopathology studies showed a myopathic pattern with ANXA11 aggregates

**Figure 4:**
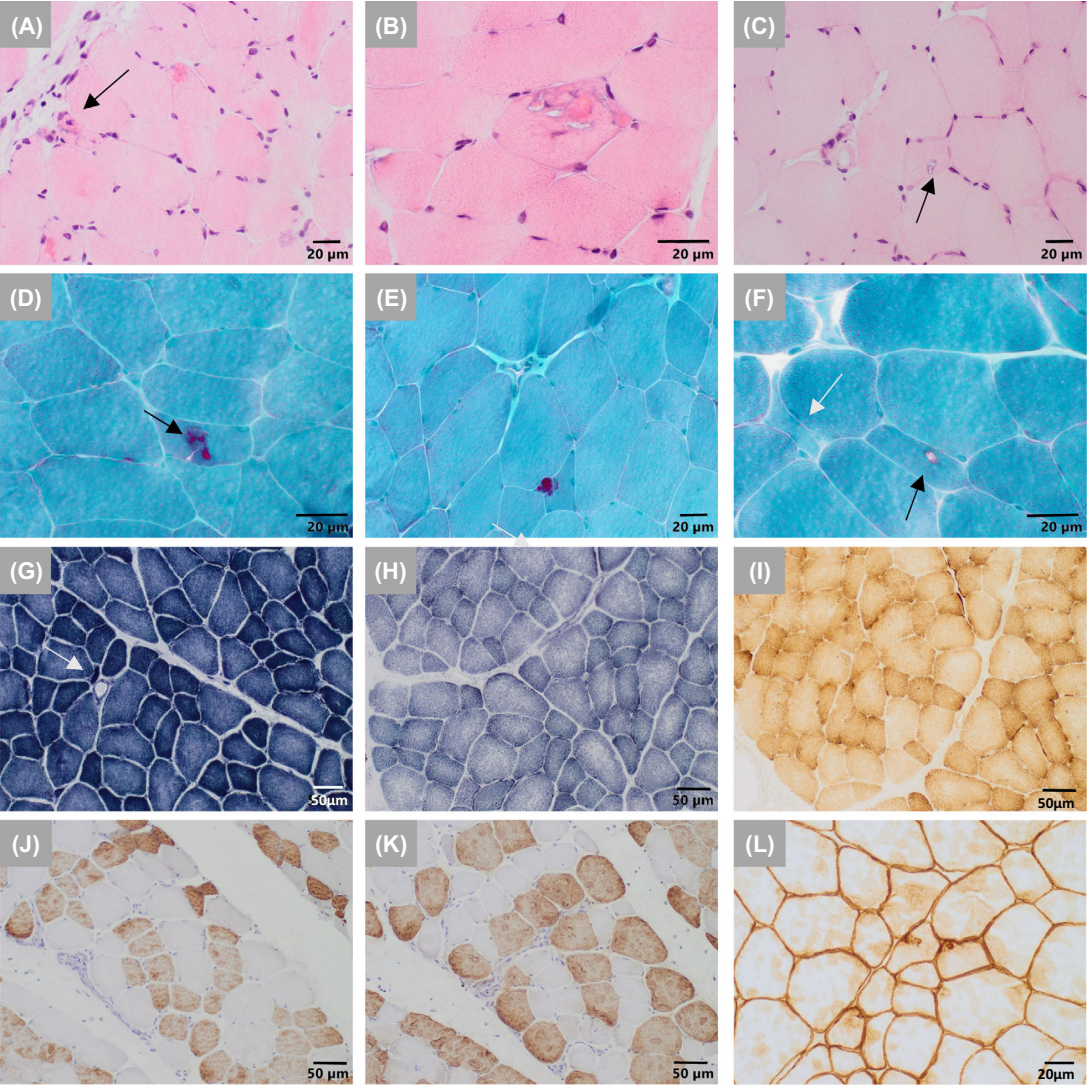
Muscle biopsy findings of the proband in early adolescence. Light microscopy studies. (A–C) The muscular biopsy showed a myopathic pattern with fiber size variability, occasionally internal myonuclei and basophilic fibers (arrow). No necrotic fibers or increased connective tissue was seen (A). Occasional muscle fibers presented dense sarcoplasmic eosinophilic aggregates with variable sizes (B). Scale bar: 20 μm. (D–F) Gomori's modified trichrome. These aggregates with Gomori's modified trichrome staining showed an intense red coloration similar to that observed in the cytoplasmic bodies. Scale bar: 20 μm. Presence of subsarcolemmal and sarcoplasmic rimmed vacuoles (C,F black arrows). These vacuoles were sometimes membrane‐lined (L, immunostaining against dystrophin). Scale bar: 20 μm. (G) NADH, (H) SDH, (I) COX. Presence of isolated angulated fibers (G, white arrow) and staining defects of the moth‐eaten intermyofibrillar pattern were identified (H and I). Scale bar: 50 μm. (J) immunostaining slow myosin, (K) immunostaining fast myosin). Pattern of distribution by types of mosaic fibers was evidenced without grouping by types of fibers. Hypotrophic fibers were of both types. Scale bar: 50 μm.

Muscle biopsy from the patient, performed in middle childhood, showed a myopathic pattern, with some internal nuclei, rimmed vacuoles, and a moth‐eaten intermyofibrillar pattern (Fig. 4A–I). These histopathological findings are similar to those identified in muscle biopsies from individuals with adult‐onset IBM.^10^, ^11^ The fibers showed an adequate distribution by fiber types with immunostaining for myosins. No grouping was identified (Fig. 4J,K). Immunohistochemical studies of muscular membrane proteins (dystrophin, sarcoglycan, beta‐dystroglycan, alpha‐dystroglycan, caveolin, dysferlin; data not shown) did not show deficits. Some of the vacuoles were covered by membrane proteins such as dystrophin (Fig. 4L).

**Figure 5:**
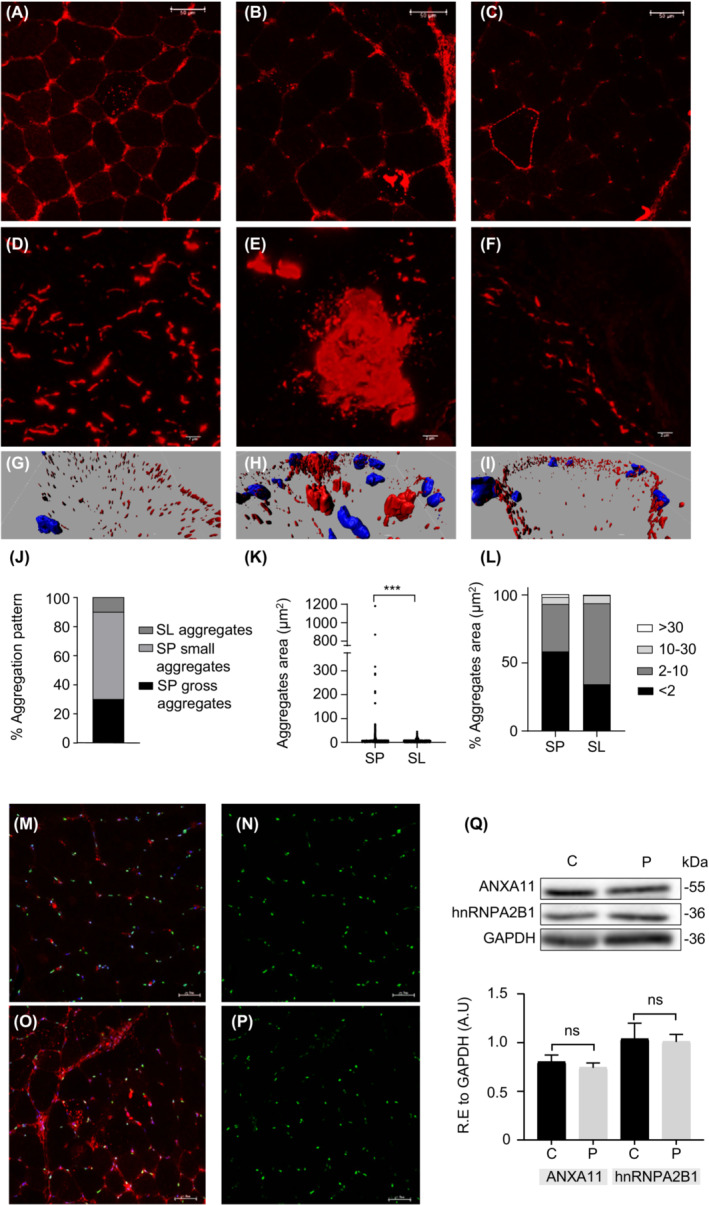
Muscle biopsy shows that ANXA11^Asp40Ile^ aggregates in muscle fibers. (A–C) Immunostaining with α‐ANXA11 revealed three different types of aggregates in muscle fibers. (A) small aggregates located in the sarcoplasm with or without relation to the sarcolemma (SP small aggregates), (B) large aggregates related or not to vacuoles (SP gross aggregates), (C) aggregates located only in the sarcolemma (SL aggregates). Scale bar: 50 μm. (D–F) Representative super‐resolution fluorescence images of ANXA11+ aggregates. (D) pearl strips with varied size and fibrillary‐shaped structures in sarcoplasm, (E) large complex structures in sarcoplasm, (F) fibrillary shapes layered as subsarcolemmal chains. Scale bar: 2 μm. (G–I) Representative confocal super‐resolution 3D images of ANXA11 aggregates showing differences in area and distribution. (J–L) Quantification of aggregates according to their pattern, area and localization. (M–P) Immunostaining with α‐ANXA11, α‐hnRNPA2B1, and DAPI stain in the pediatric healthy control (M,N) and patient (O,P). (Q) Western blot analysis and quantification of the soluble fraction of muscle protein extracts using α‐ANXA11, α‐hnRNPA2B1, and α‐GAPDH [Values are means ± SEM: ANXA11 control (C) vs. patient (P): 0.79 ± 0.07 vs. 0.72 ± 0.04, *p*: 0.441; hnRNPA2B1 C vs. P: 1.03 ± 0.16 vs. 0.92 ± 0.12, *p*: 0.598]. Two‐tailed Student's *t*‐test was used for comparisons against control (ns: not significant). SL, sarcolemma; SP, sarcoplasm.

ANXA11 immunostaining of muscle biopsy revealed additional validation of the *ANXA11* p.Asp40Ile pathogenicity. The most striking finding with respect to the histopathological descriptions of the individuals with adult‐onset IBM^10^, ^11^ was the detection of ANXA11 signal in the sarcoplasm and sarcolemma of the fibers and three different types of aggregates according to their size and location. Seventy‐one out of 1887 muscle fibers had ANXA11+ aggregates representing 3.7%. They were distributed as follows: 30% were large aggregates related or not to vacuoles, 60% were small aggregates located in the sarcoplasm with or without relation to the sarcolemma, and 10% were located only in the sarcolemma (Fig. 5A–C,J). Super‐resolution confocal images revealed ANXA11 aggregates shaped like pearl strips with varied sizes or large complex structures in the sarcoplasm (Fig. 5D,E,G,H), and as layered subsarcolemmal chains (Fig. 5F,I). In addition, quantification of the aggregates area revealed significant differences between sarcoplasm and sarcolemma. The aggregate area ranged from <0.1 to 1000 μm^2^ (Fig. 5K).

Taking into account the clinical similarities between our patient and *HNRNPA2B1*‐associated oculopharyngeal muscular dystrophy,^35^ we colabeled control and patient muscles using α‐ANXA11 and α‐hnRNPA2B1. We found no colocalization of ANXA11 and hnRNPA2B1, and absence of hnRNPA2B1 positive inclusions (Fig. 5M–P). hnRNPA2B1 was located at the nuclei of muscle fibers, which showed equal signals among samples (Fig. 5N,P). Western blot analysis of protein soluble fraction from muscle showed no difference in the levels of ANXA11 or hnRNPA2B1 (Fig. 5Q). Besides, colabeling experiments of ANXA11 with the SQSTM1 protein that aggregates in ANXA11 proteinopathies,^10^ showed colocalization in sarcoplasmic inclusions in muscle fibers thus supporting common mechanisms (Fig. S1).

**Figure 6:**
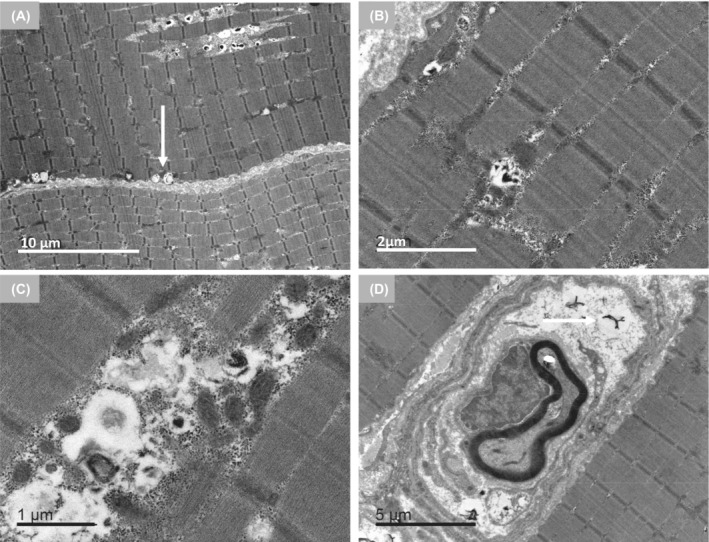
Ultrastructural findings associated with the *ANXA11* variant p.Asp40Ile. Longitudinal section of muscle fibers. (A) Subsarcolemmal electrodense structures in small vacuoles (arrow). Scale bar: 10 μm. (B,C) These osmophilic structures were as well identified inside the sarcomere forming part of the autophagic vacuole with other debris elements. Scale bar: 2 and 1 μm respectively. (D) Normal mitochondria and unspecific lipid droplets are identified in the periphery. Isolated nerve fillets with similar electrodense structures were observed (arrow). Scale bar: 5 μm.

To learn more about ANXA11 aggregates, we performed electron microscopy studies of the patient's muscle biopsy. In muscle fibers, we found small subsarcolemmal osmophilic structures (Fig. 6A) and vacuoles attached or not to the membrane with electron‐dense material similar to myelin‐like debris and remnant organelles. These vacuoles were located between the sarcomeres, distorting the intermyofibrillar pattern. Unspecific lipid and mitochondrial droplets of normal size and appearance were identified at the periphery of the vacuoles (Fig. 6B,C). Osmophilical structures with similar characteristics were observed in nerves thus providing circumstantial evidence of possible nerve pathology (Fig. 6D). Outside the vacuoles normal myofibrils were present. No sarcoplasmic tubulefilamentous inclusions were observed. The ultrastructural study revealed numerous autophagic vacuoles but no intranuclear inclusions, a typical finding in oculopharyngeal muscular dystrophy.^36^ This absence could be explained by the early onset of the disease and the patient's age at the time of the biopsy.

### ANXA11 p.Asp40Ile increases protein aggregation propensity and impacts the ability to undergo LLPS in vitro

**Figure 7:**
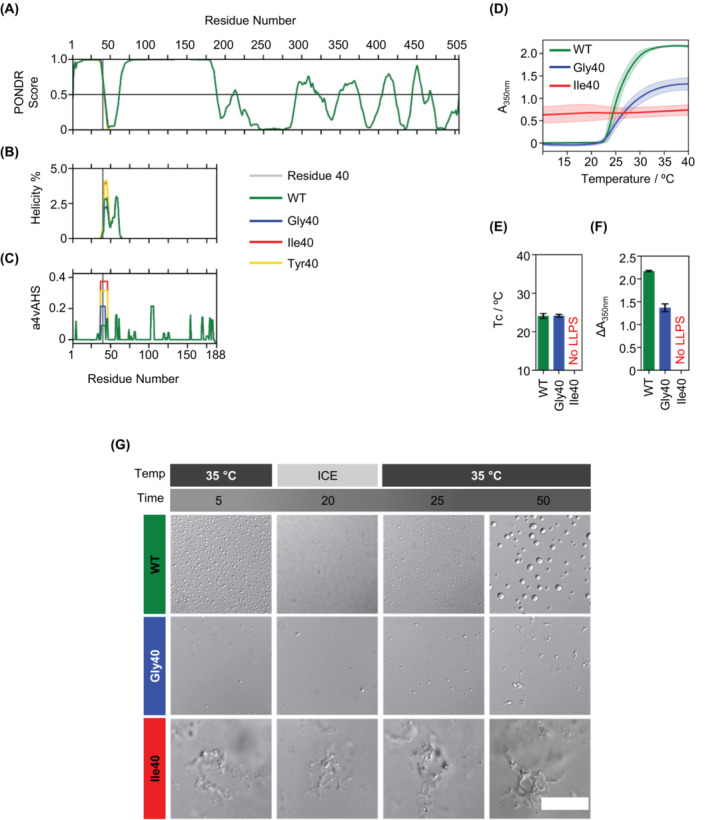
ANXA11^Asp40Ile^ has a higher aggregation propensity and does not undergo in vitro LLPS. (A–C) In silico sequence analysis of four different ANXA11 constructs (WT, Gly40, Ile40, and Tyr40). (A) Disorder size prediction of the full‐length protein using the PONDR VL‐XT algorithm. (B) Percentage helicity prediction of the intrinsically disordered domain (IDD) according to Agadir. (C) Aggregation propensity of the IDD calculated using Aggrescan (a4vAHS). The vertical gray line indicates the mutated amino acid position (Asp40 in WT). (D–F) In vitro LLPS separation study of the three ANXA11 constructs (WT, Gly40, and Ile40). All samples contain 15 μM of protein in 20 mM HEPES buffer, with 500 mM NaCl, 1 mM TCEP, 0.05% NaN3 at pH 7.4. (D) Apparent absorbance measurements at 350 nm in function of temperature as the average and standard deviation of three independent measurements for each construct. (E) Calculated cloud temperatures (Tc/°C). (F) Increase in apparent absorbance upon LLPS. For E and F, values could not be calculated for the Ile40 mutant because it did not undergo LLPS in the tested conditions. (G) Differential interference contrast microscopy images of in vitro LLPS of WT, Gly40, and Ile40 upon a temperature (Temp) cycle from 35°C to ice and back to 35°C. The time (min) since sample preparation is also indicated at the top. Scale bar: 20 μm.

Since ALS‐causing *ANXA11* variants affecting the IDD increase protein aggregation propensity,^6^ we first carried out a computational analysis of the structure and aggregation propensity^30^, ^31^, ^32^ of ANXA11^WT^, ANXA11^Asp40Ile^, ANXA11^Asp40Tyr^ and ANXA11^Asp40Gly^ full‐length proteins. As previously reported,^6^ we found that the ANXA11^WT^ low‐complexity domain is predicted to be completely disordered, except for a 50 amino acid long region next to the Asp40 position. The comparison of ANXA11 allelic variants showed a slight increase in helical propensity predicted for these residues in ANXA11^Asp40Ile^ and ANXA11^Asp40Tyr^, compared to ANXA11^Asp40Gly^ and ANXA11^WT^. Additionally, Aggrescan predicts an increase in aggregation propensity, relative to ANXA11^WT^, of the region of sequence close to the mutation site in the case of ANXA11^Asp40Gly^, which was higher in ANXA11^Asp40Tyr^ and even higher in ANXA11^Asp40Ile^ (Fig. 7A–C).

Next, we investigated whether AnxA11^p.Asp40Ile^ affects liquid droplet formation similarly to ANXA11^Asp40Gly^.^6^ LLPS assays were performed using purified ANXA11^WT^, ANXA11^Asp40Gly^, and ANXA11^Asp40Ile^ full‐length recombinant proteins. As previously described, we found that ANXA11^WT^ undergoes LLPS at high temperature (Fig. 7D–G) while ANXA11^Asp40Gly^ undergoes LLPS at the same temperature although to a lesser extent (the maximum absorbance reached is half that of ANXA11^WT^, and there are fewer and smaller droplets by DIC microscopy). ANXA11^WT^ droplets formation could be reversed by lowering the temperature (4°C for 30 min), whereas ANXA11^Asp40Gly^ droplets remained abnormally aggregated under this condition.^6^ In the case of the patient's variant, our results showed that ANXA11^Asp40Ile^ does not undergo LLPS under the same conditions but instead forms aggregates over the entire temperature range and those are not temperature reversible (Fig. 7G). Taken together, all these data argue that the p.Asp40Ile variant has a greater deleterious impact on ANXA11 solubility and ability to undergo LLPS than other pathogenic substitutions of *ANXA11* at the Asp40 position.

### The p.Asp40Ile variant of ANXA11 is associated with reduced basal levels of ANXA11 and hnRNPA2B1 and alters the dynamics of SG

**Figure 8:**
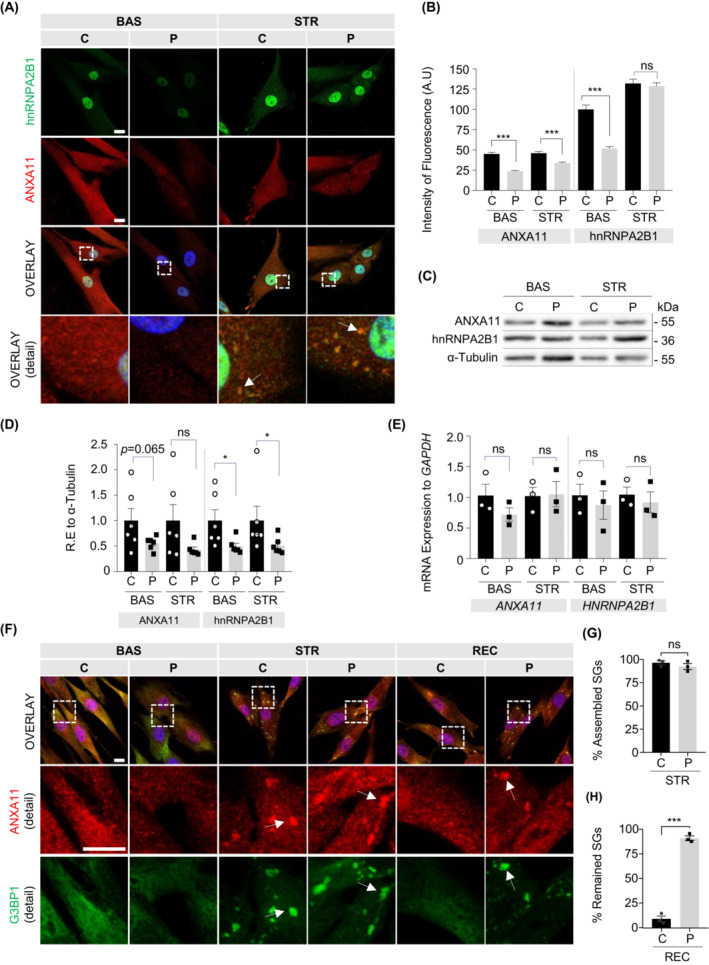
ANXA11^Asp40Ile^ and hnRNPA2B1 levels are decreased in patient fibroblasts and affect the stress granule dynamics impairing the disassembly process. (A) Immunostaining of endogenous ANXA11 (red) and hnRNPA2B1 (green) in control and patient fibroblasts in basal and after stress (0.5 mM sodium arsenite for 1 h) conditions. Nuclei were stained with DAPI. Scale bar: 25 μm. White arrows indicate the stress granules SGs. (B) Quantification of endogenous ANXA11 and hnRNPA2B1 fluorescence intensity (50 cells per condition were quantified for signal intensity from two independent experiments). (C,D) Western blot analysis (*n* = 6) and quantification of ANXA11 and hnRNPA2B1 using α‐Tubulin for data normalization in control and patient fibroblasts [Baseline: ANXA11 mean ± SEM, control (C) vs. patient (P), 1.0 ± 0.24 vs. 0.55 ± 0.05 *p*: 0.065; Stress: 1.0 ± 0.31 vs. 0.42 ± 0.05 *p*: 0.164; hnRNPA2B1 C vs. P basal 1.0 ± 0.21 vs. 0.49 ± 0.06 *p*: 0.023, stress 1.0 ± 0.28 vs. 0.51 ± 0.07 *p*: 0.041]. (E) *ANXA11* and *HNRNPA2B1* mRNA quantification by RT‐qPCR to *GAPDH* in basal and stress conditions. The samples are triplicates from three independent experiments. (F) Fluorescence images of endogenous ANXA11 (red) and G3BP1 (green) of control and patient fibroblasts in basal, stress, and recovery conditions. Nuclei were stained with DAPI. White arrows show SGs. (G) Percentage of assembled positive ANXA11+ and G3BP1+ SGs in control and patient fibroblasts. (H) Percentage of remaining positive ANXA11+ and G3BP1+ SGs after recovery in control and patient fibroblasts (100 cells per condition were quantified for SGs quantification from three independent experiments). BAS, Basal; REC, Recovery; STR, Stress. Values are means ± SEM. Mann–Whitney test was used for comparisons against control in each condition (*p < 0.05, ***p < 0.001, ns: not significant). Scale bars: 25 μm.

Since ANXA11 and hnRNPA2B1 participate in the formation and dynamics of SGs,^5^, ^37^ we experimentally assessed the SGs dynamics. We used fibroblasts from the patient and healthy controls, which we treated with 0.5 mM sodium arsenite for 1 h. First, we quantified by immunostaining with α‐ANXA11 and α‐hnRNPA2B1 the signal of these proteins in fibroblasts. The patient showed a significantly lower ANXA11 signal in both conditions compared to the control (basal *p*: <0.0001, stress *p*: <0.0001, recovery *p*: <0.0001) whilst the hnRNPA2B1 signal was reduced exclusively under basal conditions (*p*: <0.0001 for basal, *p*: 0.5923 for stress) (Fig. 8A,B). Although the levels of both proteins were decreased in the patient's fibroblasts, we observed the formation of SGs, where ANXA11 and hnRNPA2B1 colocalized (Fig. 8A, arrows). Quantification of the soluble fraction of fibroblast protein extracts by Western blot showed the tendency for soluble ANXA11 levels to decrease under basal conditions while soluble hnRNPA2B1 levels were significantly decrease under both basal and stress conditions (Fig. 8C,D, Fig. S2). The study of *ANXA11* and *HNRNPA2B1* mRNAs by qRT‐PCR revealed no changes (ANXA11, basal *p*: 0.400; stress *p*: 0.999; *HNRNPA2B1*, *p*: 0.700 for basal, *p*: 0.400 for stress) (Fig. 8E). To further investigate SGs defects we immunostained G3BP Stress Granule Assembly Factor 1 (G3BP1), which is a *bona fide* component of cytoplasmic SGs.^38^ As in control fibroblasts, ANXA11 strongly colocalized with G3BP1 in the SGs of the patient's fibroblasts (Fig. 8F). We found no differences among samples in SGs assemble (*p*: 0.358) (Fig. 8G). After 1.5 h of recovery condition, only 9% of SGs were retained in control fibroblasts, whilst more than 90% of SGs did not disassemble in the patient's fibroblasts (*p*: <0.0001) (Fig. 8H). This defect in SG disassembly has been reported in other *ANXA11*‐related ALS‐linked variants.^6^ Our results demonstrate that the new *ANXA11* p.Asp40Ile variant has an impact on ANXA11 and hnRNPA2B1 biology and causes defective dynamics of SGs by altering their disassembly and clearance process.

## Discussion

The study of the new *ANXA11* variant p.Asp40Ile in a patient with a unique childhood‐onset muscular dystrophy phenotype has demonstrated that disorders associated with *ANXA11* Asp40 allelic variants have a common pathophysiology.

We propose that Aspartate 40 is essential for the stability of ANXA11 against aggregation and importantly, it is the severity of Asp40 substitution that could underlie the pleiotropic expression. First, p.Asp40Ile is predicted to enhance the aggregation propensity of ANXA11 to a greater extent than other pathological changes affecting this residue; second, recombinant ANXA11^p.Asp40Ile^ showed abnormal phase separation and higher aggregation propensity than ANXA11^p.Asp40Gly^ that causes ALS; third, the patient's fibroblasts had lower expression of ANXA11 and hnRNPA2B1 both involved in SG, and defective SG dynamics, and fourth, the patient's muscle tissue showed complex ANXA11 aggregation patterns in both the sarcoplasm and sarcolemma of the muscle fibers.

p.Asp40Ile increases the aggregation propensity of ANXA11 and alters the phase separation that is essential for the assembly of membraneless organelles such as SGs. Our qualitative and quantitative measurements of ANXA11 aggregates revealed wide variability in their shape, area, and distribution in the muscle fiber thus suggesting different impacts of the p.Asp40Ile clinical variant on the multifunctionality of ANXA11 and especially of its IDD. When mutations occur in IDRs, the lack of a structure makes it challenging to rationalize their effects on the interactions and functions of the protein. Moreover, defects in LLPS could affect not only the formation of SGs but other membraneless organelles, which are commonly found in the nucleus and cytoplasm of cells playing a wide variety of functions,^39^ which can be affected in the patients.

Clinically, this study shows that pathologic changes in ANXA11 can cause a phenotype reminiscent of oculopharyngeal muscular dystrophy but with a strikingly earlier age of onset and a faster progression. This new phenotype has a unique set of clinical and histopathological features that make it recognizable: ptosis, ophthalmoplegia, dysphagia, respiratory failure, progressive muscle weakness, autophagic vacuoles, and electron‐dense aggregates. Moreover, the phenotype and pathologic features of the muscle in our patient were similar to that recently described in patients carrying frameshift *HNRNPA2B1* variants.^35^ Since no variants were detected in the *HNRNPA2B1* gene of the patient and the mRNA levels in his fibroblasts were normal, we propose that the observed decrease in hnRNPA2B1 protein in fibroblasts could be related to the decrease in its partner ANXA11, which would facilitate its degradation. Indeed, p.Asp40Ile causes the reduction of soluble ANXA11 levels in fibroblasts. Perturbation of the ANXA11 disordered domain by this variant could affect the half‐life of the protein in the cell as has already been described for other variants affecting N‐terminal disordered segments.^40^ Although more work is needed, these results support the hypothesis of hnRNPA2B1 haploinsufficiency as a possible pathophysiological link between the clinical features observed in our patient and carriers of *HNRNPA2B1* mutations. On the other hand, in ALS cases the reported change in hnRNPA2B1 was an aspartate substitution affecting the IDD^13^ thus showing the deleterious impact of the Aspartate substitutions on disordered domains and their pleiotropic expression.

Overall, our findings broaden the phenotypic spectrum of *ANXA11*‐related conditions that includes ALS, FTD and both adult‐ and pediatric‐onset progressive myopathies. Noteworthy is the striking difference in the age of onset between the individual we describe and individuals with the other two variants of Asp40 (5th‐6th decade). We propose that muscle is more vulnerable to higher‐impact changes of Asp40 in ANXA11 causing early‐onset symptoms and primary muscle involvement, whereas a lower‐impact variant is expressed as a late‐onset disorder with motor neuron involvement phenotype, as can occur in other conditions such as muscle sarcopenia. Indeed, the primary involvement of muscle and the neuromuscular junction in ALS, although controversial, has been supported by reports of muscle pathological changes independent of motor neuron degeneration occurring before the ALS‐onset and evidence in animal models showing that muscle is primarily affected.^41^, ^42^ Finally, ANXA11 forms part of the landscape of pleiotropic proteins related to human disease. Knowledge of this pleiotropy would provide a mechanistic framework to explain the genetic and functional architecture of *ANXA11* spectrum disorders caused by aspartate 40 variants, and new clues for designing treatments to inhibit ANXA11 abnormal aggregation or by activating protein clearance mechanisms.

## Author Contributions

JH, DNdB, FP, and AN contributed to the study concept and design. JH, DNdB, FP, and AN were involved in drafting the manuscript and/or figures. DNdB, JEE, LCG, CO, and AN contributed to clinical management, analysis, and clinical data ascertainment of the patient. JO, CGC, AC, MR, CBJ, JH, and XS contributed to acquisition of experimental data. All authors have contributed to the revision of the manuscript for important intellectual content and have approved the final version of this paper. All authors involved in experimental studies were responsible of the statistical analyses. DNdB and JO are joint first authors. FP, AN, and JH are joint last authors.

## Conflict of Interest

Xavier Salvatella is a founder and board member of Nuage Therapeutics. The other authors report no disclosures.

## Supporting information


**Table S1.** Detailed information about primary and secondary antibodies used in immunohistochemistry, immunofluorescence, and western blot techniques
**Table S2**. List of primers used for Sanger sequencing validation of c.118_119delGAinsAT and qPCR.
**Table S3**. Clinical features of the individual with the ANXA11 variant c.118_119delGAinsAT/p.Asp40Ile.
**Table S4**. In silico pathogenicity predictions and variant frequency for ANXA11 c.118_119delGAinsAT/p.Asp40Il.
**Figure S1**. Double immunofluorescence studies for α‐SQSTM1 in green, α‐ANXA11 in red, and the nuclei stained with DAPI (blue) in the patient muscle.
**Figure S2**. Western Blot membranes of ANXA11 and hnRNPA2B1.

## Data Availability

Data are available on reasonable request.
