# Supplementary material for: Common pathophysiology for ANXA11 disorders caused by aspartate 40 variants
Source: Ann Clin Transl Neurol. 2023 Jan 18;10(3):408–25. doi: 10.1002/acn3.51731 (PMC10014011; doi:10.1002/acn3.51731)
Supplement: Supplementary file 1 — Table S1. Detailed information about primary and secondary antibodies used in immunohistochemistry, immunofluorescence, and western blot techniques Table S2. List of primers used for Sanger sequencing validation of c.118_119delGAinsAT and qPCR. Table S3. Clinical features of the individual with the ANXA11 variant c.118_119delGAinsAT/p.Asp40Ile. Table S4. In silico pathogenicity predictions and variant frequency for ANXA11 c.118_119delGAinsAT/p.Asp40Il. Figure S1. Double immunofluorescence studies for α‐SQSTM1 in green, α‐ANXA11 in red, and the nuclei stained with DAPI (blue) in the patient muscle. Figure S2. Western Blot membranes of ANXA11 and hnRNPA2B1. [file ACN3-10-408-s001.docx]

**Common pathophysiology for ANXA11 disorders caused by aspartate 40 variants**

Daniel Natera-de Benito, MD, PhD, Jonathan Olival, MSc, Carla Garcia-Cabau, MSc, Cristina Jou, MD, Mònica Roldan, PhD, Anna Codina, MSc, Jessica Expósito-Escudero, MD, Cristina Batlle, PhD, Laura Carrera-García, MD, Carlos Ortez, MD, Xavier Salvatella, PhD, Francesc Palau, MD, PhD, Andrés Nascimento, MD, Janet Hoenicka, PhD

Supplementary File contents: Table S1, Table S2, Table S3, Table S4, Fig. S1, and Fig. S2

| **IMMUNOHISTOCHEMISTRY** | | | |
| --- | --- | --- | --- |
| **Antibody** | **Clonality** | **Host** | **Source** |
| Dystrophin (Dys1) Rod domain | MAb^a^ | Mouse | Novocastra, Leica Biosystems, Newcastle, UK |
| **IMMUNOFLUORESCENCE** | | | |
| **Antibody** | **Clonality** | **Host** | **Source** |
| Myosin Heavy Chain Fast | MAb | Mouse | Novocastra, Leica Biosystems, Newcastle, UK |
| Myosin Heavy Chain Slow | MAb | Mouse | Novocastra, Leica Biosystems, Newcastle, UK |
| SQSTM1 (p62) | MAb | Mouse | Santa Cruz Biotechnology, Dallas, TX, USA |
| ANXA11 | PAb^b^ | Rabbit | Sigma- Aldrich , Sant Louis, MO, USA |
| hnRNPA2B1 | MAb | Mouse | Santa Cruz Biotechnology, Dallas, TX, USA |
| G3BP1 | MAb | Mouse | Santa Cruz Biotechnology, Dallas, TX, USA |
| **WESTERN BLOT** | | | |
| **Antibody** | **Clonality** | **Host** | **Source** |
| ANXA11 | PAb | Rabbit | Santa Cruz Biotechnology, Dallas, TX, USA |
| hnRNPA2B1 | MAb | Mouse | Santa Cruz Biotechnology, Dallas, TX, USA |
| G3BP1 | MAb | Mouse | Santa Cruz Biotechnology, Dallas, TX, USA |
| Alpha Tubulin | MAb | Mouse | Sigma-Aldrich, St. Louis, MO, USA |
| **SECONDARY ANTIBODIES** | | | |
| **Antibody** | **Clonality** | **Host** | **Source** |
| α-Mouse Alexa Fluor 488 | PAb | Goat | Thermo Fisher Scientific, Inc |
| α-Mouse Alexa Fluor 488 | PAb | Donkey | Thermo Fisher Scientific, Inc |
| α-Rabbit Alexa Fluor 594 | PAb | Goat | Thermo Fisher Scientific, Inc |
| α-Rabbit Alexa Fluor 594 | PAb | Donkey | Thermo Fisher Scientific, Inc |
| α-Mouse HRP Conjugated | PAb | Goat | Thermo Fisher Scientific, Inc |
| α-Rabbit HRP Conjugated | PAb | Goat | Jackson Immuno Research, PA, USA |
| a MAb: Monoclonal Antibody  b PAb: Polyclonal Antibody | | | |

**Supplementary Table S1. Detailed information about primary and secondary antibodies used in immunohistochemistry, immunofluorescence, and western blot techniques**

| **Sanger Sequencing** | | | | | |
| --- | --- | --- | --- | --- | --- |
| **Gene** | **Accession** | **Forward Primer (5’-3’)** | **Reverse Primer (5’-3’)** | **Amplification gDNA^a^** | **Product Size** |
| *ANXA11* | NM_145868.2 | TCATGTGGTCTGTGGTGTCC | GGCAGAAGCCTGATGAAAA | chr10:80170635-80171058 | 424 bp |
| **RT-PCR** | | | | | |
| **Gene** | **Accession** | **Forward Primer (5’-3’)** | **Reverse Primer (5’-3’)** | **Amplification cDNA^b^** | **Product Size** |
| *ANXA11* | NM_145868.2 | CCCACCTGGTAGCAGTTTTC | GGCTGGGGTATTCTTGAGAC | c.1165-1302 | 137 bp |
| *HNRNPA2B1* | NM_002137.4 | TGTAGCAAGAGAGGAATCTGG | ACGATTTTATCCACAGGATCATG | c.251-473 | 222 bp |
| a Genomic DNA start and end nucleotide amplification sites  b Coding DNA start and end nucleotide amplification sites | | | | | |

**Supplementary Table S2. List of primers used for Sanger sequencing validation of c.118_119delGAinsAT and qPCR**

**Supplementary Table S3. Clinical features of the individual with the *ANXA11* variant c.118_119delGAinsAT/p.Asp40Ile.**

| **Sex** | **Ethnicity** | ***ANXA11***  **Variant^a^** | **Age at Onset** | **First Symptoms** | **Age at Last Examination** | **Pattern of Muscle Weakness** | **CK^b^** | **Histological Findings** |
| --- | --- | --- | --- | --- | --- | --- | --- | --- |
| Male | Caucasian | c.118_119delGAinsAT p.(Asp40Ile) | 3 years | Ptosis | 20 years | Ptosis and ophthalmoparesis from 5 years old, and from the age of 14 years, progressive facial, proximal, and distal weakness, more pronounced in the lower extremities, as well as dysphonia and dysphagia | 950 IU^c^ | Fiber size variation  Internal nuclei  Rimmed vacuoles  Protein inclusions in sarcoplasmic and subsarcolemmal localization |

a Nucleotide numbering is according to the reference MANE transcript *ANXA11* NM_145868.2

b CK: creatine kinase

c IU: international units.

**Supplementary Table S4. *In silico* pathogenicity predictions and variant frequency for *ANXA11* c.118_119delGAinsAT/p.Asp40Il**

| **PATHOGENICITY PREDICTORS** | | | | | | | | | | | | | | |
| --- | --- | --- | --- | --- | --- | --- | --- | --- | --- | --- | --- | --- | --- | --- |
| **CADD^a^** | **Varsome (7^th^ November, 2022)** | | **MutationTaster^c^** | | | **SIFT^d^** | | **PROVEAN^e^** | | | **PolyPhen2^f^** | | **FATHMM^g^** | |
|  | **Verdict** | **ACMG^b^ Rules** | **Prediction** | | **Score** | **Prediction** | **Score** | **Prediction** | **Score** | | **Prediction** | **Score** | **Prediction** | **Score** |
| 23.9 | Pathogenic | PS2 Strong, PS3 Strong, PM1 Moderate,  PM2 Moderate, PM5 Moderate, PP3 Supporting | Damaging | | 0.999 | Damaging | 0.02 | Neutral | -1.09 | | Probably damaging | 0.996 | Tolerated | 4.55 |
| **VARIANT FREQUENCY** | | | | | | | | | | | | | | |
| **gnomAD^h^** | | | | **ClinVar^i^** | | | | | | **CSVS^j^** | | | | |
| Variant Not Found | | | | Variant Not Found | | | | | | Variant Not Found | | | | |
| *In silico* pathogenicity prediction was carried out according to the reference MANE transcript *ANXA11* NM_145868.2   1. CADD. Combined Annotation Dependent Depletion. The variant is pathogenic above 15 ^9^. 2. ACMG. American College of Medical Genetics. Varsome categories: Pathogenic, Likely Pathogenic, VUS (Variant of Uncertain Significance9, Likely Benign, Benign ^2^. 3. MutationTaster. Scores close to 1 indicates a high 'security' of the damaging or polymorphism prediction ^4^. 4. SIFT. Sorting Intolerant from Tolerant. Scores range between 0 (pathogenic) and 1 (benign) ^5^. 5. PROVEAN. Protein Variation Effect Analyzer. If the PROVEAN score is equal to or below a predefined threshold (-2.5), the protein variant is predicted to have a "deleterious" effect. If the PROVEAN score is above the threshold, the variant is predicted to have a "neutral" effect ^6^. 6. PolyPhen2. Polymorphism Phenotyping v2. Scores range between 0 (benign) and 1 (pathogenic)^7^. 7. FATHMM. Functional Analysis through Hidden Markov Models. Scores range -16.13 (benign) and 10.64 (pathogenic) ^8^. 8. gnomAD. Genome Aggregation Database ^10^. 9. ClinVar. Categories: Benign, Likely benign, Uncertain significance, Likely pathogenic, Pathogenic, drug response, association risk factor, conflicting data from submitters, other, not provide ^11^. 10. CSVS. Collaborative Spanish Variant Server ^12^. | | | | | | | | | | | | | | |

**
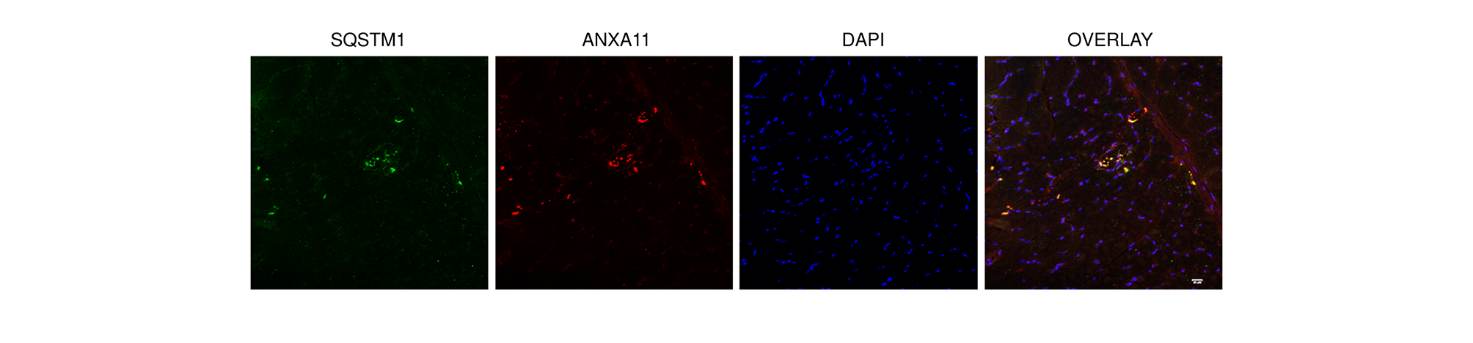
**

**Supplementary Fig. S1. Colocalization of SQSTM1 and ANXA11 in the aggregates of the patient muscle fibers**

**Supplementary Fig. S1. Double immunofluorescence studies for α-SQSTM1 in green, α-ANXA11 in red, and the nuclei stained with DAPI (blue) in the patient muscle**. It showed colocalization in the aggregates indicating activation of autophagy pathways in the patient. Scale bar: 20 μm.

STR

BAS

STR

BAS

STR

BAS


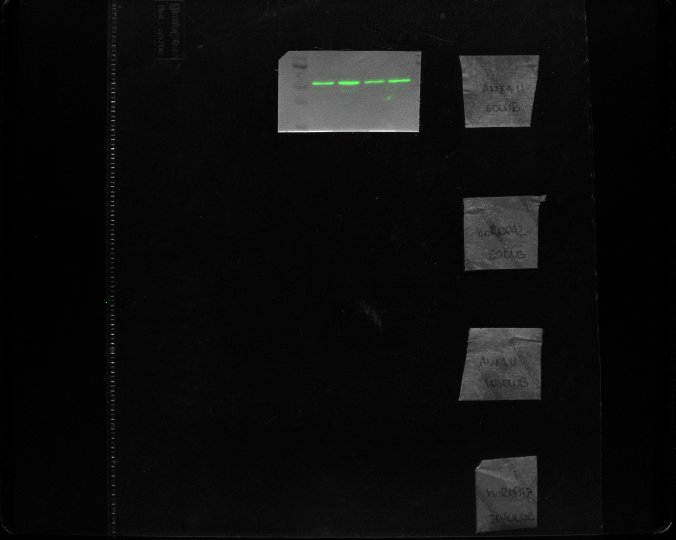


C P C P

kDa

C P C P

75

37

kDa

C P C P

kDa


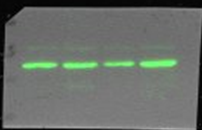

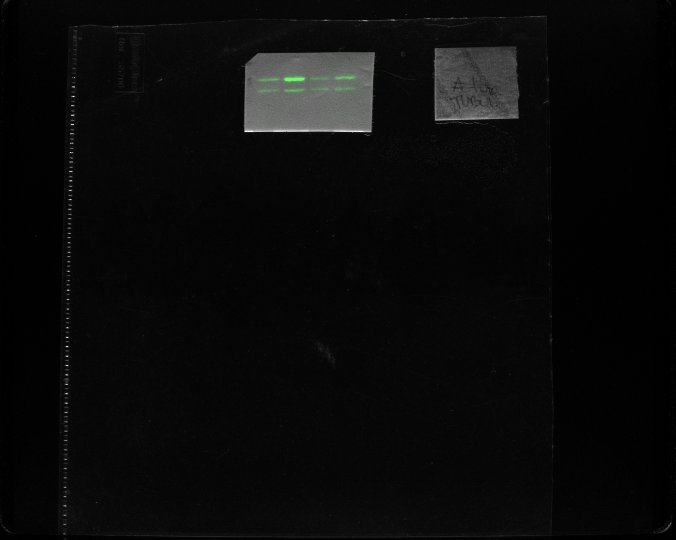


75

75

ANXA11

α-Tubulin

50

50

hnRNPA2B1

50

37

37

**Supplementary Fig. S2. Western Blot membranes of ANXA11 and hnRNPA2B1**

Uncut Western Blot membranes showing ANXA11, hnRNPA2B1, and α-Tubulin protein bands (green) of control and patient fibroblasts in basal and stress conditions. C: control, P: patient, BAS: basal, STR: stress.

**References**

1. Li H, Durbin R. Fast and accurate long-read alignment with Burrows-Wheeler transform. *Bioinformatics* 2010;26:589-95. doi: 10.1093/bioinformatics/btp698

2. Kopanos C, Tsiolkas V, Kouris A, et al. VarSome: the human genomic variant search engine. *Bioinformatics* 2019;35:1978-80. doi: 10.1093/bioinformatics/bty897

3. Richards S, Aziz N, Bale S, et al. Standards and guidelines for the interpretation of sequence variants: A joint consensus recommendation of the American College of Medical Genetics and Genomics and the Association for Molecular Pathology. *Genetics in Medicine* 2015;17:405-24. doi: 10.1038/gim.2015.30

4. Schwarz JM, Cooper DN, Schuelke M, et al. Mutationtaster2: Mutation prediction for the deep-sequencing age. *Nature Methods* 2014;11:361-62. doi: 10.1038/nmeth.2890

5. Sim NL, Kumar P, Hu J, et al. SIFT web server: Predicting effects of amino acid substitutions on proteins. *Nucleic Acids Research* 2012;40:W452-W57. doi: 10.1093/nar/gks539

6. Choi Y, Chan AP. PROVEAN web server: A tool to predict the functional effect of amino acid substitutions and indels. *Bioinformatics* 2015;31:2745-47. doi: 10.1093/bioinformatics/btv195

7. Adzhubei IA, Schmidt S, Peshkin L, et al. A method and server for predicting damaging missense mutations. *Nature Methods* 2010;7:248-49. doi: 10.1038/nmeth0410-248

8. Shihab HA, Rogers MF, Gough J, et al. An integrative approach to predicting the functional effects of non-coding and coding sequence variation. *Bioinformatics* 2015;31:1536-43. doi: 10.1093/bioinformatics/btv009

9. Rentzsch P, Witten D, Cooper GM, et al. CADD: Predicting the deleteriousness of variants throughout the human genome. *Nucleic Acids Research* 2019;47:D886-D94. doi: 10.1093/nar/gky1016

10. Karczewski KJ, Francioli LC, Tiao G, et al. The mutational constraint spectrum quantified from variation in 141,456 humans. *Nature* 2020;581:434-43. doi: 10.1038/s41586-020-2308-7

11. Landrum MJ, Lee JM, Benson M, et al. ClinVar: Improving access to variant interpretations and supporting evidence. *Nucleic Acids Research* 2018;46:D1062-D67. doi: 10.1093/nar/gkx1153

12. Peña-Chilet M, Roldán G, Perez-Florido J, et al. CSVS, a crowdsourcing database of the Spanish population genetic variability. *Nucleic Acids Research* 2021;49:D1130-D37. doi: 10.1093/nar/gkaa794

13. Dubowitz V, Sewry C, Oldfors A. Muscle Biopsy: A Practical Approach. 2013 [published Online First: 4th editio]

14. Romero, Obradovic, Dunker K. Sequence Data Analysis for Long Disordered Regions Prediction in the Calcineurin Family. *Genome Inform Ser Workshop Genome Inform* 1997;8:110-24. [published Online First: 1997/01/01]

15. Munoz V, Serrano L. Elucidating the folding problem of helical peptides using empirical parameters. III. Temperature and pH dependence. *J Mol Biol* 1995;245(3):297-308. doi: 10.1006/jmbi.1994.0024 [published Online First: 1995/01/20]

16. Conchillo-Solé O, de Groot NS, Avilés FX, et al. AGGRESCAN: A server for the prediction and evaluation of "hot spots" of aggregation in polypeptides. *BMC Bioinformatics* 2007:65. doi: 10.1186/1471-2105-8-65

17. Alberti S, Saha S, Woodruff JB, et al. A User's Guide for Phase Separation Assays with Purified Proteins. *Journal of Molecular Biology* 2018;430:4806-20. doi: 10.1016/j.jmb.2018.06.038
